# Synthesis and Antifungal Activity of Novel Triazole Compounds Containing Piperazine Moiety

**DOI:** 10.3390/molecules190811333

**Published:** 2014-07-31

**Authors:** Yanwei Wang, Kehan Xu, Guojing Bai, Lei Huang, Qiuye Wu, Weihua Pan, Shichong Yu

**Affiliations:** 1 Department of Organic Chemistry, School of Pharmacy, Second Military Medical University, 325Guohe Road, Shanghai 200433, China; 2 Shanghai Key Laboratory of Molecular Medical Mycology, Department of Dermatology, Shanghai Changzheng Hospital, Second Military Medical University, 415 Fengyang Road, Shanghai 200003, China; 3 Department of Pharmacy, No. 422 Hospital of PLA, 40 Haibin No. 3 Road, Zhanjiang 524000, China

**Keywords:** antifungal, triazole, synthesis, piperazine

## Abstract

Design and synthesis of triazole library antifungal agents having piperazine side chains, analogues to fluconazole were documented. The synthesis highlighted utilization of the click chemistry on the basis of the active site of the cytochrome P450 14α-demethylase (CYP51). Their structures were characterized by ^1^H-NMR, ^13^C-NMR, MS and IR. The influences of piperazine moiety on *in vitro* antifungal activities of all the target compounds were evaluated against eight human pathogenic fungi.

## 1. Introduction

**Figure 1:**
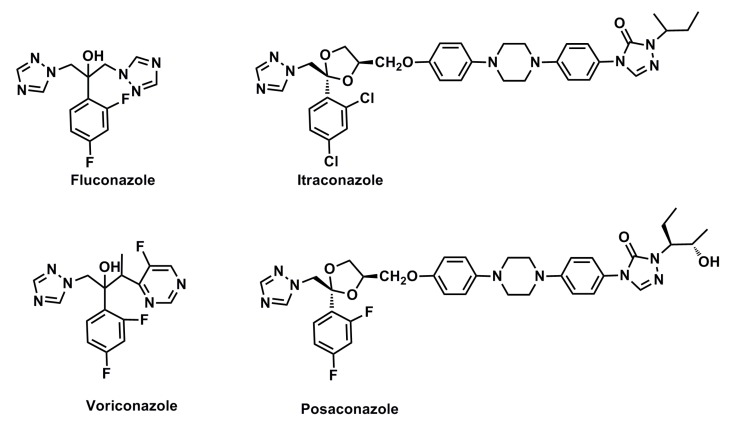
Triazole antifungal agents used in clinical therapy.

Triazole compounds have been specially paid increasing attention because of their extensively medicinal applications in antimicrobial agent’s particularly antifungal therapy, and a large number of predominant triazole drugs have been successfully developed and prevalently used in the treatment of various microbial infections for many years [1]. Azoles (fluconazole, itraconazole, voriconazole, and posaconazole, Figure 1) are important antifungal drugs for the treatment of invasive fungal infections (IFIs), which continue to be a major cause of morbidity and mortality in immunocompromised orseverely ill patients [2]. However, fluconazole is not effective against invasive aspergillosis and has suffered severe drug resistance [3,4]. The increasing frequency of fungal infections and development of resistance to the current treatment highlight the need for development of new triazole derivatives possessing broader antifungal spectra and higher therapeutic indexes.

Azole antifungals act by competitive inhibition of CYP51, the enzyme that catalyzes the oxidative removal of the 14α-methyl group of lanosterol to give Δ^14,15^-desaturated intermediates in ergosterol biosynthesis [5]. In general, the active site of CYP51 for ligand binding can be divided into four subsites: a coordination bond with iron of the heme group, the hydrophilic H-bonding region, the hydrophobic region, and the narrow hydrophobic cleft formed by the residues in the helix B'-meander 1 loop and N-terminus of helix I [6]. These compounds target the biosynthesis of ergosterol by inhibiting the cytochrome P450-dependent lanosterol 14α-demethylase (Erg11p, CYP51), encoded by the ERG11 gene, resulting in accumulation of toxic methylsterols inmembranes that may culminate in fungi static effect or fungal death [7].

Literature precedents [8,9] revealed a pharmacophore of antifungal triazoles, which contained a triazole ring linking to a dihalophenyl ring through a two carbon chain. In addition, the carbon alpha to the phenyl ring bears a hydroxyl group. Itraconazole and posaconazole which containing the group of piperazine. We intended to alter the side chains to find potent systemic antifungal compounds with a broad antifungal spectrum and less potential to develop resistance. In our previous works [10,11,12,13,14,15,16], many studies on the structure-activity relationships (SAR) of antifungal azoles have been developed, and these studies have led to new compounds endowed with better biological and pharmacological properties.

**Figure 2:**
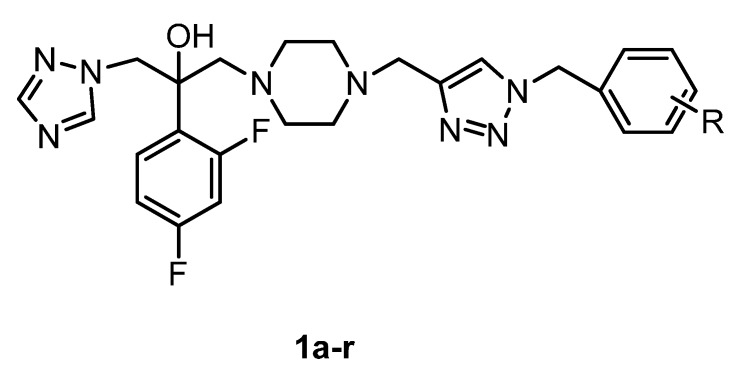
Generic structure of the designed fluconazole analogues.

According to the above results, we designed a series of 1-(1*H*-1,2,4-triazole-1-yl)-2-(2,4-difluorophenyl)-3-substituted-2-propanols (**1a**–**r** Figure 2) containing a triazole ring, a difluorophenyl group, a hydroxyl group and a side chain containing piperazine group. In our design, we systematically altered the structure of fluconazole as a platform and tried to insert the 1,2,3-triazole group into the side chain.

## 2. Results and Discussion

### 2.1. Chemistry

**Scheme 1:**
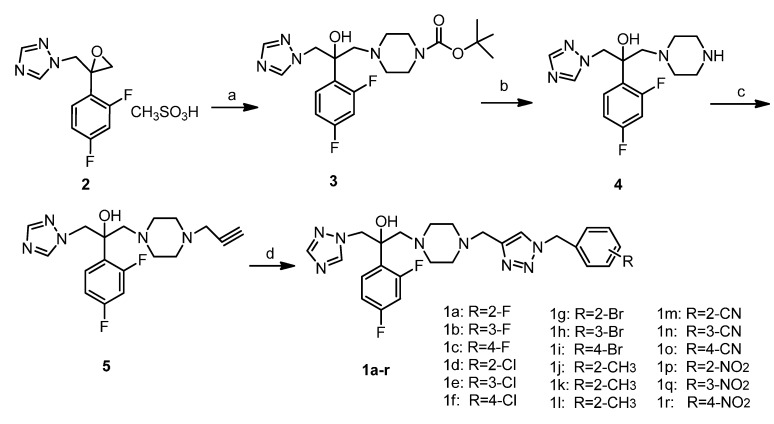
Synthesis of the target compounds **1a**–**r**. Conditions: (**a**) Et_3_N, *N*-Boc-piperazine, EtOH, reflux, 6 h, 80%; (**b**) CF_3_COOH, room temperature, 1 h, 87%; (**c**) propargyl bromide, KI, K_2_CO_3_, CH_3_CN, room temperature, 5–6 h, 72%; (**d**) NaN_3_, substituted benzyl bromide, DMSO, CuSO_4_.5H_2_O, sodium ascorbate, rt, 12 h, 60%–70%.

Target compounds **1a**–**r** were synthesized according to a very efficient and straightforward synthetic route outlined in Scheme 1. Compound **3** was synthesized by ring-open reaction of oxirane **2** with *N*-Boc-piperazine and simultaneous Boc-deprotection was accomplished by treatment with F_3_CCOOH to furnish compound **4**. *N*-alkylation was effective in the presence of KI, K_2_CO_3_ and propargyl bromide in acetonitrile at room temperature to secure compound **5**. The target compounds were achieved by using click reaction [17] with variously substituted benzyl azides.

### 2.2. Pharmacology

The *in vitro* antifungal activities of all the target compounds were evaluated against eight human pathogenic fungi, *Candida albicans 14053 (C.alb.14053)*, *Candida albicans 20352 (C.alb.20352)*, *Candida parapsilosis (C. Par.)*, *Cryptococcus neoformans (C.neo.)*, *Candida glabrata (C. gla.)*, *Aspergillus fumigates (A.fum.)*, *Trichophyton rubrum (T.rub.)*, *Microsporum gypseum (M. gyp.)*, which are often encountered clinically, and were compared with itraconazole (ICZ), voriconazole (VCZ) and fluconazole (FCZ). All of the above eight human pathogenic fungi were provided by Shanghai Changzheng Hospital; Fluconazole (FCZ), itraconazole (ICZ) and voriconazole (VCZ) served as the positive control and were obtained from their respective manufacturers.

**Table 1:** Antifungal activities of the target compounds *in vitro* (MIC_80_ μg/mL).

Compd	*C. alb14053*	*C. alb. 20352*	*C. par.*	*C. neo.*	*C. gla.*	*A. fum.*	*T. rub.*	*M. gyp.*
**1a**	2	8	4	8	>64	>64	8	4
**1b**	1	4	4	4	4	>64	4	16
**1c**	1	2	8	2	4	>64	8	8
**1d**	0.25	0.5	1	1	16	>64	2	8
**1e**	1	1	1	1	16	>64	1	4
**1f**	0.5	0.5	1	1	>64	>64	1	1
**1g**	0.5	0.5	1	1	16	>64	1	0.5
**1h**	0.5	0.5	1	0.5	32	>64	1	0.5
**1i**	0.25	0.5	0.5	0.5	>64	>64	1	0.5
**1j**	1	2	4	4	>64	>64	4	0.25
**1k**	64	64	4	4	>64	>64	4	0.25
**1l**	1	4	4	0.5	64	>64	1	0.25
**1m**	4	16	16	16	>64	>64	16	16
**1n**	8	8	16	8	>64	>64	16	4
**1o**	8	8	16	8	>64	>64	16	2
**1p**	2	4	8	4	>64	>64	4	2
**1q**	1	8	8	8	>64	>64	4	2
**1r**	2	8	8	4	64	>64	4	0.25
ICZ	0.0625	0.0625	0.03125	0.0156	0.5	2	0.125	4
VCZ	0.03125	0.0625	0.03125	0.0156	0.5	0.25	0.0625	0.25
FCZ	1	0.5	0.5	0.5	2	>64	4	64

Abbreviations: *C.alb.14053*, *Candida albicans14053*; *C.alb.20352*, *Candida albicans20352*; *C. par.*, *Candida parapsilosis*; *C.neo.*, *Cryptococcus neoformans*; *C. gla.*, *Candida glabrata*; *A.fum.*, *Aspergillusfumigates*; *T.rub.*, *Trichophytonrubrum*; *M. gyp.*, *Microsporum gypseum*. ICZ, Itraconazole; VCZ, Voriconazole; FCZ, Fluconazole.

The *in vitro* minimal inhibitory concentrations (MICs) of the compounds were determined by the micro-broth dilution method in 96-well microtest plates according to the methods defined by the National Committee for Clinical Laboratory Standards (NCCLS) [18]. The MIC_80_ was defined as the first well with an approximate 80% reduction in growth compared to the growth of the drug-free well. For assays, the title compounds to be tested were dissolved in dimethyl sulfoxide (DMSO), serially diluted in growth medium, inoculated and incubated at 35 °C Growth MIC was determined at 24 h for *C. alb.* and at 72 h for *C. neo.* The *in vitro* antifungal activities are summarized in Table 1. The MIC values (in μg/mL) are presented against different pathogenic fungi, in comparison with ICZ, VCZ and FCZ.

The results of antifungal activities *in vitro* showed that all the 18 target compounds (**1a**–**r**) were active against nearly all fungi tested to some extent except against *A. fum.* and *C. gla.* The MIC_80_ value of compound **1d** and **1i** is 4 times lower than that of FCZ against *C. alb. 14053 in vitro* (with the MIC_80_ value of 0.25 μg/mL). The MIC_80_ value of compound **1j**, **1k**, **1l** and **1r** is 128 times lower than that of FCZ against *M. gyp. in vitro*, and the same as VCZ against *M. gyp. in vitro* (with the MIC_80_ value of 0.25 μg/mL). However, most of the target compounds’ antifugal activities were not as good as the ICZ and VCZ.

## 3. Experimental Section

### 3.1. General Procedures

^1^H and spectra were recorded in CDCl_3_ unless otherwise indicated with a Bruker AC-300P spectrometer, using TMS as internal standard. ESI mass spectra were performed on an API-3000 LC-MS spectrometer. The solvents and reagents were used as received or dried prior to use as needed.

*Tert-butyl-4-(2-(2,4-difluorophenyl)-2-hydroxy-3-(1H-1,2,4-triazol-1-yl)propyl)piperazine-1-carboxylate* (**3**): A mixture of compound **2** (16.65 g, 0.05 mol), CH_3_CH_2_OH (300 mL) and Et_3_N (25 mL), *N*-Boc-piperazine (13.96 g, 0.075 mol) was stirred and reﬂuxed for 6 h. The reaction was monitored by TLC. After filtration, the filtrate was evaporated under reduced pressure. Water was added to the residue, extracted with ethylacetate twice, combinate the organic layer, washed with saturated NaHCO_3_ and NaCl solution twice, dried over anhydrous Na_2_SO_4_ and evaporated to get compound **3** (16.93 g, 80%).

*1-(1H-1,2,4-Triazole-1-yl)-2-(2,4-difluorophenyl)-3-(piperazin-1-yl)-2-propanol* (**4**): A mixture of compound **3** (4.23 g, 0.01 mol), Trifluoroacetic acid (11.4 g, 0.1 mol) was stirred at room temperature for 30 min. The reaction was monitored by TLC. After reaction, most of the trifluoroacetic acid was removed in a vacuum. Column chromatography of the residue afforded compound **4** as yellow oil (DCM/CH_3_OH, 20:1–10:1, 2.81 g, 87%).

*1-(1H-1,2,4-Triazole-1-yl)-2-(2,4-difluorophenyl)-3-(4-(prop-2-yn-1-yl)piperazin-1-yl)-2-propanol* (**5**): A mixture of compound **4** (3.23 g, 0.01 mol), propargyl bromide (2.36 g, 0.02 mol), KI (166 mg, 0.001 mol), K_2_CO_3_ (3.45 g, 0.025 mol), and CH_3_CN (50 mL) was stirred at room temperature for 6 h. The reaction was monitored by TLC. After reaction, we filtrated off the solid and washed with CH_3_CN. The filtrate was concentrated in a vacuum. Column chromatography of the residue afforded compound **5** as a brown oil (DCM/CH_3_OH, 20:1–10:1, 2.60 g, 72%).

### 3.2. General Procedure for the Preparation of the Compounds **1a**–**r**

A mixture of NaN_3_ (100 mg, 1.4 mmol), 2-fluorobenzyl bromide (200 mg, 1.2 mmol) and DMSO (15 mL) was stirred at room temperature for 6 h. Then the compound **5** (217 mg, 0.6 mmol) was added, sodium ascorbate (20 mg), CuSO_4_^.^5H_2_O (25 mg) and H_2_O (1 mL) was added, then stirred at room temperature for another 2 h. The reaction was monitored by TLC, then NH_3_^.^H_2_O was added carefully and extracted with ethylacetate. The organic layer was acidificated with dilute hydrochloric acid, then the aqueous layer was adjusted pH about 7.0 by saturation sodium bicarbonate, then extracted with ethylacetate, washed with water, NaHCO_3_ and NaCl, and dried with Na_2_SO_4_. It was then concentrated in a vacuum. Column chromatography of the residue afforded compound **1a** as yellow oil. (212 mg, 69%).^1^H-NMR (300 MHz, CDCl_3_) δ: 8.17 (s, 1H), 7.81 (s, 1H), 7.58–7.55 (m, 2H), 7.46–7.34 (m, 2H), 7.32–7.27 (m, 1H), 7.19–7.09 (m, 1H), 6.85–6.78 (m, 2H), 5.58 (s, 2H), 4.53 (s, 2H), 3.62 (s, 2H), 3.10–2.63 (m, 2H), 2.41–2.27 (m, 8H); ^13^C NMR (75 MHz, CDCl_3_) δ: 163.7, 158.7, 151.3, 147.5, 144.1, 139.1, 132.5, 130.1, 129.9, 129.8, 129.7, 127.7, 124.3, 123.7, 119.1, 118.5, 114.1, 111.4, 104.1, 71.7, 62.1, 56.3, 54.1, 53.1, 51.3; IR(KBr): 3380, 2923, 2821, 2100, 1615, 1498, 1458, 1273, 1137, 1048, 1015, 966, 852, 675, 616, 515 cm^−1^; ESI-MS, *m/z* calcd. for C_25_H_27_F_3_N_8_O 512.2, found [M+H]^+^ 513.5.

The target compounds **1b**–**r** were synthesized by the same operation procedure of the compound **1a**.

## 4. Conclusions

In summary, a novel series of antifungal agents have been designed and synthesized by chemical methods. *In vitro* antifungal activity assay indicated that most of the compounds showed antifungal activities against both systemic pathogenic fungi. Several compounds show high *in vitro* antifungal activity with a broad spectrum, which were valuable for further evaluation.

## Supplementary Materials

Supplementary materials can be accessed at: http://www.mdpi.com/1420-3049/19/8/11333/s1.

## Supplementary Files

Supplementary File 1

## References

[1] Gadhave P.P., Dighe N.S., Pattan S.R., Deotarse P., Musmade D.S., Shete R. (2010). Current biological and synthetic profile of triazoles: A review. Annals Biol. Res..

[2] Groll A.H., Lumb J. (2012). New developments in invasive fungal disease. Future Microbiol..

[3] Casalinuovo I.A., di Francesco P., Garaci E. (2004). Fluconazole resistance in Candida albicans: A review of mechanisms. Eur. Rev. Med. Pharmacol..

[4] Hoffman H.L., Ernst E.J., Klepser M.E. (2000). Novel triazole antifungal agents. Expert Opin. Inv. Drug.

[5] Aoyama Y., Yoshida Y., Sato R. (1984). Yeast cytochrome P-450 catalyzing lanosterol 14 alpha-demethylation. II. Lanosterol metabolism by purified P-450(14)DM and by intact microsomes. J. Biol. Chem..

[6] Sheng C.Q., Zhang W.N., Ji H.T., Song Y.L., Zhang M., Zhou Y.J., Lu J.G., Zhu J. (2004). Design, synthesis and antifungal activity of novel triazole derivatives. Chin. Chem. Lett..

[7] Kelly S., Arnoldi A., Kelly D. (1993). Molecular genetic analysis of azole antifungal mode of action. Biochem. Soc. Trans..

[8] Sheng C.Q., Zhang W.N., Ji H.T., Zhang M., Song Y.L., Xu H., Zhu J., Miao Z.Y., Jiang Q.F., Yao J.Z. (2006). Structure-based optimization of azole antifungal agents by CoMFA, CoMSIA, and molecular docking. J. Med. Chem..

[9] Boyle F.T., Gilman D.J., Gravestock M.B., Wardleworth J.M. (1988). Synthesis and structure-activity relationships of a novel antifungal agent, ICI 195,739. Ann. NY Acad. Sci..

[10] Zou Y., Yu S., Li R., Zhao Q., Li X., Wu M., Huang T., Chai X., Hu H., Wu Q. (2014). Synthesis, antifungal activities and molecular docking studies of novel 2-(2,4-difluorophenyl)-2-hydroxy-3-(1*H*-1,2,4-triazol-1-yl) propyl dithiocarbamates. Eur. J. Med. Chem..

[11] Yu S., Wang L., Wang Y., Song Y., Cao Y., Jiang Y., Sun Q., Wu Q. (2013). Molecular docking, design, synthesis and antifungal activity study of novel triazole derivatives containing the 1,2,3-triazole group. RSC Advan..

[12] Yu S., Chai X., Wang N., Cui H., Zhao Q., Hu H., Zou Y., Sun Q., Wu Q. (2013). Synthesis and antifungal activity of the novel triazole compounds. Med. Chem. Commun..

[13] Wang N., Chai X., Chen Y., Zhang L., Li W., Gao Y., Bi Y., Yu S., Meng Q. (2013). Synthesis, antifungal activity, and molecular docking studies of novel triazole derivatives. Med. Chem..

[14] Chai X., Yu S., Jiang Y., Zou Y., Wu Q., Zhang D., Jiang Y., Cao Y., Sun Q. (2012). Design, synthesis, and biological evaluation of novel 1,2,4-triazole derivatives as antifungal agent. Arch. Pharm. Res..

[15] Wang B.G., Yu S.C., Chai X.Y., Yan Y.Z., Hu H.G., Wu Q.Y. (2011). Design synthesis and biological evaluation of 3-substituted triazole derivatives. Chin. Chem. Lett..

[16] Yu S., Chai X., Hu H., Yan Y., Guan Z., Zou Y., Sun Q., Wu Q. (2010). Synthesis and antifungal evaluation of novel triazole derivatives as inhibitors of cytochrome P450 14 alpha-demethylase. Eur. J. Med. Chem..

[17] Zhang X.J., Li H.Y., You L.F., Tang Y., Hsung R.P. (2006). Copper salt-catalyzed azide-[3 + 2] cycloadditions of ynamides and bis-ynamides. Adv. Synth. Catal..

[18] National Committee for Clinical Laboratory Standards (2002). Reference Method for Broth Dilution Antifungal Susceptibility Testing of Yeasts Approved standard.

