# Supplementary material for: Synthesis and Antifungal Activity of Novel Triazole Compounds Containing Piperazine Moiety
Source: Molecules. 2014 Jul 31;19(8):11333–40. doi: 10.3390/molecules190811333 (PMC6271785; doi:10.3390/molecules190811333)
Supplement: Supplementary File 1 [file molecules-19-11333-s001.pdf]

Supplementary Information

The title compounds 1b-r were characterized as follows:

1-(1H-1,2,4-Triazol-1-yl)-2-(2,4-difluorophenyl)-3-(4-((1-(3-fluorobenzyl)-1H-1,2,3-triazol-4-yl)methyl) piperazin-1-yl)-2-propanol (**1b**). Oil, yield, 62%; ^{1}H-NMR (300 MHz, $CDCl_3$) δ: 8.22 (s, 1H), 7.87 (s, 1H), 7.64–7.53 (m, 2H), 7.38–7.28 (m, 2H), 7.17–7.04 (m, 2H), 6.97–6.80 (m, 2H), 5.55 (s, 2H), 4.59 (s, 2H), 4.27 (s, 2H), 3.09–2.66 (m, 2H), 2.26–2.03 (m, 8H); ^{13}C-NMR (75 MHz, $CDCl_3$) δ: 162.7, 158.5, 151.1, 147.2, 144.5, 139.3, 132.3, 130.2, 129.7, 129.7, 129.6, 127.6, 124.4, 123.5, 119.3, 118.7, 114.3, 111.3, 104.5, 71.9, 62.3, 56.3, 54.0, 53.3, 51.1; IR(KBr): 3383, 2922, 2825, 2101, 1617, 1499, 1455, 1271, 1135, 1047, 1019, 963, 851, 673, 615, 513 cm^{-1}; ESI-MS, *m/z* calcd. for $C_{25}H_{27}F_3N_8O$ 512.2, found $[M+H]^+$ 513.7.

1-(1H-1,2,4-Triazol-1-yl)-2-(2,4-difluorophenyl)-3-(4-((1-(4-fluorobenzyl)-1H-1,2,3-triazol-4-yl)methyl) piperazin-1-yl)-2-propanol (**1c**). Oil, yield, 65%; ^{1}H-NMR (300 MHz, $CDCl_3$) δ: 8.12 (s, 1H), 7.78 (s, 1H), 7.68–7.51 (m, 2H), 7.39–7.25 (m, 2H), 7.19–7.02 (m, 2H), 6.97–6.82 (m, 2H), 5.50 (s, 2H), 4.52 (s, 2H), 3.64 (s, 2H), 3.17–2.61 (m, 2H), 2.61–2.22 (m, 8H); ^{13}C-NMR (75 MHz, $CDCl_3$) δ: 164.7, 158.8, 151.3, 147.3, 144.3, 139.1, 132.1, 130.1, 129.8, 129.7, 129.6, 127.6, 124.5, 123.7, 119.3, 118.8, 114.1, 111.4, 104.2, 71.8, 62.1, 56.1, 54.1, 53.1, 51.1; IR(KBr): 3381, 2925, 2828, 2104, 1619, 1497, 1458, 1274, 1138, 1049, 1017, 964, 854, 676, 618, 516 cm^{-1}; ESI-MS, *m/z* calcd. for $C_{25}H_{27}F_3N_8O$ 512.2, found $[M+H]^+$ 513.6.

1-(1H-1,2,4-Triazol-1-yl)-2-(2,4-difluorophenyl)-3-(4-((1-(2-chlorobenzyl)-1H-1,2,3-triazol-4-yl)methyl) piperazin-1-yl)-2-propanol (**1d**). Oil, yield, 66%; ^{1}H-NMR (300 MHz, $CDCl_3$) δ: 8.17 (s, 1H), 7.81 (s, 1H), 7.65–7.52 (m, 1H), 7.47–7.45 (m, 2H), 7.39–7.21 (m, 3H), 6.86–6.39 (m, 2H), 5.66 (s, 2H), 4.54 (s, 2H),3.53(s, 2H), 3.11–2.64 (m, 2H), 2.43–2.31 (m, 8H); ^{13}C-NMR (75 MHz, $CDCl_3$) δ: 162.7, 158.7, 151.0, 147.1, 144.7, 139.3, 132.5, 130.4, 129.7, 129.7, 129.6, 127.6, 124.5, 122.7, 119.1, 118.8, 114.1, 111.4, 104.3, 71.9, 62.2, 56.3, 54.2, 53.0, 51.4; IR(KBr): 3380, 2924, 2852, 1613, 1523, 1498, 1460, 1421, 1347, 1271, 1207, 1137, 1048, 1008, 964, 855, 724, 679, 653, 515 cm^{-1}; ESI-MS, *m/z* calcd. for $C_{25}H_{27}ClF_2N_8O$ 528.2, found $[M+H]^+$ 529.8.

*1-(1H-1,2,4-Triazol-1-yl)-2-(2,4-difluorophenyl)-3-(4-((1-(3-chlorobenzyl)-1H-1,2,3-triazol-4-yl)methyl) piperazin-1-yl)-2-propanol (*1**e**). Oil, yield, 69%; ^{1}H-NMR (300 MHz, $CDCl_3$) δ: 8.12 (s, 1H), 7.80 (s, 1H), 7.65–7.45 (m, 2H), 7.36–7.24 (m, 3H), 7.17–7.08 (m, 1H), 6.86–6.76 (m, 2H), 5.49 (s, 2H), 4.54 (s, 2H), 3.77 (s,2H), 3.12–2.67 (m, 2H), 2.60–2.48 (m, 8H); ^{13}C-NMR (75 MHz, $CDCl_3$) δ: 162.7, 158.7, 151.0, 147.1, 144.7, 139.3, 132.5, 132.4, 130.4, 130.2, 127.6, 124.5, 124.4, 122.7, 119.1, 114.1, 111.7, 104.3, 104.2, 71.9, 62.2, 56.3, 54.2, 53.0, 51.4; IR(KBr): 3383, 2926, 2855, 1616, 1525, 1499, 1464, 1423, 1349, 1273, 1209, 1139, 1043, 1008, 966, 857, 728, 674, 656, 513 cm^{-1}; ESI-MS, *m/z* calcd. for $C_{25}H_{27}ClF_2N_8O$ 528.2, found $[M+H]^+$ 529.7.

1-(1H-1,2,4-Triazol-1-yl)-2-(2,4-difluorophenyl)-3-(4-((1-(4-chlorobenzyl)-1H-1,2,3-triazol-4-yl)methyl) piperazin-1-yl)-2-propanol (**1f**). Oil, yield, 65%; ^{1}H-NMR (300 MHz, $CDCl_3$) δ: 8.10 (s, 1H), 7.82 (s, 1H), 7.68–7.60 (m, 2H), 7.42–7.29 (m, 2H), 7.12–6.96 (m, 2H), 6.85–6.69 (m, 2H), 5.55 (s, 2H), 4.58 (s, 2H), 3.68 (s,2H), 2.27–2.03 (m, 2H), 2.41–2.27 (m, 8H); ^{13}C-NMR (75 MHz, $CDCl_3$) δ: 163.7,

158.8, 151.1, 147.3, 144.9, 139.5, 132.1, 132.1, 130.2, 130.2, 129.6, 124.7, 124.2, 122.9, 119.3, 114.5, 111.9, 104.5, 104.3, 71.7, 62.0, 56.1, 54.3, 53.1, 51.3; IR(KBr): 3384, 2927, 2856, 1617, 1526, 1495, 1465, 1424, 1347, 1274, 1207, 1137, 1042, 1008, 965, 856, 727, 673, 655, 515 cm^{-1}; ESI-MS, *m/z* calcd. for $C_{25}H_{27}ClF_2N_8O$ 528.2, found $[M+H]^+$ 529.6..

1-(1H-1,2,4-Triazol-1-yl)-2-(2,4-difluorophenyl)-3-(4-((1-(2-bromobenzyl)-1H-1,2,3-triazol-4-yl)methyl) piperazin-1-yl)-2-propanol (**1g**). Oil, yield, 67%; ^{1}H-NMR (300 MHz, $CDCl_3$) δ: 8.17 (s, 1H), 7.78 (s, 1H), 7.57–7.41 (m, 2H), 7.34–7.21 (m, 3H), 7.20–7.17 (m, 1H), 6.86–6.75 (m, 2H), 5.64 (s, 2H), 4.52 (s, 2H), 3.63 (s,2H), 3.80–2.66 (m, 2H), 2.51–2.38 (m, 8H); ^{13}C-NMR (75 MHz, $CDCl_3$) δ: 162.7, 159.0, 151.1, 144.5, 143.6, 134.3, 132.2, 129.9, 129.5, 129.3, 129.3, 126.0, 122.8, 111.5, 111.4, 104.5, 104.2, 103.7, 72.4, 62.1, 56.2, 54.0, 53.9, 53.4, 52.7; IR(KBr): 3383, 2921, 2820, 2097, 1613, 1495, 1455, 1272, 1133, 1045, 1008, 964, 850, 679, 614, 515 cm^{-1}; ESI-MS, *m/z* calcd. for $C_{25}H_{27}BrF_2N_8O$ 572.1, found $[M+H]^+$ 573.4.

1-(1H-1,2,4-Triazol-1-yl)-2-(2,4-difluorophenyl)-3-(4-((1-(3-bromobenzyl)-1H-1,2,3-triazol-4-yl)methyl) piperazin-1-yl)-2-propanol (**1h**). Oil, yield, 65%; ^{1}H-NMR (300 MHz, $CDCl_3$) δ: 8.11 (s, 1H), 7.53 (s, 1H), 7.59–7.50 (m, 2H), 7.42–7.39 (m, 2H), 7.36–7.24 (m, 2H), 6.86–6.75 (m, 2H), 5.61 (s, 2H), 4.58 (s, 2H), 3.92 (s, 2H), 3.09–2.73 (m, 2H), 2.55–2.25 (m, 8H); ^{13}C-NMR (75 MHz, $CDCl_3$) δ: 162.8, 158.9, 151.0, 144.6, 143.8, 134.4, 132.3, 129.8, 129.7, 129.4, 129.3, 126.1, 122.9, 111.7, 111.5, 104.6, 104.3, 103.9, 72.2, 62.2, 56.3, 54.1, 53.8, 53.5, 52.8; IR(KBr): 3387, 2924, 2820, 2099, 1616, 1498, 1458, 1272, 1137, 1049, 1011, 964, 850, 679, 616, 515 cm^{-1}; ESI-MS, *m/z* calcd. for $C_{25}H_{27}BrF_2N_8O$ 572.1, found $[M+H]^+$ 573.3.

1-(1H-1,2,4-Triazol-1-yl)-2-(2,4-difluorophenyl)-3-(4-((1-(4-bromobenzyl)-1H-1,2,3-triazol-4-yl)methyl) piperazin-1-yl)-2-propanol (**1i**). Oil, yield, 61%; ^{1}H-NMR (300 MHz, $CDCl_3$) δ: 8.14 (s, 1H), 7.77 (s, 1H), 7.68–7.60 (m, 2H), 7.56–7.46 (m, 3H), 7.20–7.10 (m, 3H), 6.83–6.74 (m, 1H), 5.44 (s, 2H), 4.51 (s, 2H), 3.64 (s, 2H), 3.07–2.62 (m, 2H), 2.61–2.21 (m, 8H); ^{13}C-NMR (75 MHz, $CDCl_3$) δ: 162.5, 158.8, 151.2, 144.4, 143.5, 134.2, 132.1, 129.8, 129.4, 129.2, 129.2, 126.1, 122.7, 111.2, 111.2, 104.7, 104.3, 103.5, 72.2, 62.0, 56.3, 54.1, 53.7, 53.1, 52.5; IR(KBr): 3386, 2923, 2821, 2098, 1615, 1497, 1457, 1271, 1136, 1047, 1010, 963, 851, 677, 615, 513 cm^{-1}; ESI-MS, *m/z* calcd. for $C_{25}H_{27}BrF_2N_8O$ 572.1, found $[M+H]^+$ 573.7.

1-(1H-1,2,4-Triazol-1-yl)-2-(2,4-difluorophenyl)-3-(4-((1-(2-methylbenzyl)-1H-1,2,3-triazol-4-yl)methyl) piperazin-1-yl)-2-propanol (**1j**). Oil, yield, 61%; ^{1}H-NMR (300 MHz, $CDCl_3$) δ: 8.13 (s, 1H), 7.79 (s, 1H), 7.57–7.49 (m, 1H), 7.33–7.24 (m, 2H), 7.22–7.13 (m, 3H), 6.85–6.76 (m, 2H), 5.46 (s, 2H), 4.53 (s, 2H), 3.75 (s, 2H), 3.10–2.66 (m, 2H), 2.62–2.44 (m, 8H), 2.34 (s, 3H); ^{13}C-NMR (75 MHz, $CDCl_3$) δ: 162.6, 158.9, 151.1, 144.4, 138.9, 134.4, 129.7, 129.6, 129.6, 129.5, 129.4, 125.1, 123.8, 111.5, 111.3, 104.5, 104.1, 103.9, 72.4, 62.1, 62.1, 56.2, 54.4, 53.2, 52.4, 21.5; IR(KBr): 3403, 2921, 2821, 1613, 1497, 1458, 1321, 1271, 1205, 1135, 1051, 1008, 963, 851, 775, 745, 675, 653, 612, 514 cm^{-1}; ESI-MS, *m/z* calcd. for $C_{26}H_{30}F_2N_8O$ 508.3, found $[M+H]^+$ 509.2.

1-(1H-1,2,4-triazol-1-yl)-2-(2,4-difluorophenyl)-3-(4-((1-(3-methylbenzyl)-1H-1,2,3-triazol-4-yl)methyl) piperazin-1-yl)-2-propanol (**1k**). Oil, yield, 72%; ^{1}H-NMR (300 MHz, $CDCl_3$) δ: 8.11 (s, 1H), 7.78

(s, 1H), 7.55–7.47 (m, 1H), 7.35–7.22 (m, 2H), 7.25–7.10 (m, 3H), 6.89–6.76 (m, 2H), 5.51 (s, 2H), 4.61 (s, 2H), 3.74 (s, 2H), 3.11–2.54 (m, 2H), 2.52–2.44 (m, 8H). 2.35 (s,3H); ^{13}C-NMR (75 MHz, $CDCl_3$) δ: 162.7, 158.9, 151.2, 144.5, 138.9, 134.2, 129.6, 129.5, 129.5, 129.4, 129.3, 125.2, 123.5, 111.6, 111.5, 104.6, 104.2, 103.8, 72.5, 62.1, 62.6, 56.1, 54.2, 53.2, 52.5, 21.4; IR(KBr): 3405, 2925, 2821, 1616, 1497, 1458, 1321, 1271, 1205, 1138, 1052, 1008, 965, 851, 778, 743, 678, 653, 616, 515 cm^{-1}; ESI-MS, *m/z* calcd. for $C_{26}H_{30}F_2N_8O$ 508.3, found $[M+H]^+$ 509.6.

1-(1H-1,2,4-Triazol-1-yl)-2-(2,4-difluorophenyl)-3-(4-((1-(4-methylbenzyl)-1H-1,2,3-triazol-4-yl)methyl) piperazin-1-yl)-2-propanol (**1l**). Oil, yield, 63%; ^{1}H-NMR (300 MHz, $CDCl_3$) δ: 8.14 (s, 1H), 7.79 (s, 1H), 7.59–7.48 (m, 1H), 7.29–7.23 (m, 2H), 7.18–7.05 (m, 3H), 6.84–6.76 (m, 2H), 5.52 (s, 2H), 4.52 (s, 2H), 3.64 (s, 2H), 3.08–2.63 (m, 2H), 2.62–2.41 (m, 8H). 2.07 (s,3H); ^{13}C-NMR (75 MHz, $CDCl_3$) δ: 162.8, 158.8, 151.1, 144.6, 138.8, 134.3, 129.6, 129.5, 129.4, 129.4, 129.3, 125.2, 123.7, 111.8, 111.7, 104.6, 104.3, 103.9, 72.6, 62.2, 62.6, 56.2, 54.3, 53.3, 52.6, 21.3; IR(KBr): 3404, 2924, 2820, 1615, 1499, 1459, 1323, 1272, 1207, 1137, 1050, 1008, 964, 850, 778, 746, 679, 654, 615, 513 cm^{-1}; ESI-MS, *m/z* calcd. for $C_{26}H_{30}F_2N_8O$ 508.3, found $[M+H]^+$ 509.4.

1-(1H-1,2,4-Triazol-1-yl)-2-(2,4-difluorophenyl)-3-(4-((1-(2-nitrobenzyl)-1H-1,2,3-triazol-4-yl)methyl) piperazin-1-yl)-2-propanol (**1m**). Oil, yield, 69%; ^{1}H-NMR (300 MHz, $CDCl_3$) δ: 8.14 (s, 1H), 7.78 (s, 1H), 7.56–7.45 (m, 3H), 7.33–7.24 (m, 1H), 7.13–7.10 (m, 2H), 6.83–6.74 (m, 2H), 5.72 (s, 2H), 4.52 (s, 2H), 3.69 (s, 2H), 3.09–2.62 (m, 2H), 2.52–2.43 (m, 8H); ^{13}C-NMR (75 MHz, $CDCl_3$) δ: 162.6, 158.8, 151.0, 148.1, 145.2, 144.8, 141.5, 129.2, 128.6, 126.2, 124.3, 122.7, 111.6, 111.5, 104.5, 104.0, 72.0, 62.2, 62.1, 58.4, 56.3, 54.1, 53.1, 52.9, 29.4; IR(KBr): 3357, 2920, 2851, 1750, 1615, 1361, 1271, 1210, 1047, 1008, 963, 852, 823, 747, 677, 612, 513 cm^{-1}; ESI-MS, *m/z* calcd. for $C_{25}H_{27}F_2N_9O_3$ 539.2, found $[M+H]^+$ 540.5.

1-(1H-1,2,4-Triazol-1-yl)-2-(2,4-difluorophenyl)-3-(4-((1-(3-nitrobenzyl)-1H-1,2,3-triazol-4-yl)methyl) piperazin-1-yl)-2-propanol (**1n**). Oil, yield, 67%; ^{1}H-NMR (300 MHz, $CDCl_3$) δ: 8.13 (s, 1H), 7.78 (s, 1H), 7.57–7.45 (m, 3H), 7.35–7.24 (m, 1H), 7.13–7.05 (m, 2H), 6.85–6.75 (m, 2H), 5.56 (s, 2H), 4.52 (s, 2H), 3.67 (s, 2H), 3.08–2.64 (m, 2H), 2.62–2.45(m, 8H); ^{13}C-NMR (75 MHz, $CDCl_3$) δ: 162.5, 158.7, 151.1, 148.2, 145.1, 144.6, 141.5, 129.3, 128.5, 126.0, 124.3, 122.8, 111.5, 111.2, 104.3, 104.0, 71.9, 62.1, 61.8, 58.3, 56.2, 54.0, 53.1, 52.8, 29.3; IR(KBr): 3359, 2920, 2851, 1753, 1616, 1363, 1272, 1212, 1047, 1008, 965, 851, 823, 746, 676, 616, 515 cm^{-1}; ESI-MS, *m/z* calcd. for $C_{25}H_{27}F_2N_9O_3$ 539.2, found $[M+H]^+$ 540.4.

1-(1H-1,2,4-Triazol-1-yl)-2-(2,4-difluorophenyl)-3-(4-((1-(4-nitrobenzyl)-1H-1,2,3-triazol-4-yl)methyl) piperazin-1-yl)-2-propanol (**1o**). Oil, yield, 69%; ^{1}H-NMR (300 MHz, $CDCl_3$) δ: 8.11 (s, 1H), 7.81 (s, 1H), 7.58–7.43 (m, 3H), 7.35–7.23 (m, 1H), 7.13–7.07 (m, 2H), 6.85–6.74 (m, 2H), 5.60 (s, 2H), 4.56 (s, 2H), 3.58 (s, 2H), 3.17–2.69 (m, 2H), 2.54–2.23 (m, 8H); ^{13}C-NMR (75 MHz, $CDCl_3$) δ: 162.7, 158.8, 151.0, 148.1, 145.1, 144.7, 141.6, 129.4, 128.6, 126.2, 124.3, 122.7, 111.6, 111.4, 104.6, 103.9, 72.0, 62.2, 61.7, 58.4, 56.3, 54.1, 53.1, 52.7, 29.7; IR(KBr): 3358, 2921, 2850, 1750, 1616, 1362, 1272, 1211, 1049, 1008, 964, 853, 824, 748, 679, 615, 515 cm^{-1}; ESI-MS, *m/z* calcd. for $C_{25}H_{27}F_2N_9O_3$ 539.2, found $[M+H]^+$ 540.2.

1-(1H-1,2,4-Triazol-1-yl)-2-(2,4-difluorophenyl)-3-(4-((1-(2-cyanobenzyl)-1H-1,2,3-triazol-4-yl)methyl) piperazin-1-yl)-2-propanol (**1p**). Oil, yield, 67%; ^{1}H-NMR (300 MHz, $CDCl_3$) δ: 8.10 (s, 1H), 7.81 (s, 1H), 7.73–7.70 (m, 2H), 7.64–7.59 (m, 1H), 7.54–7.37 (m, 3H), 6.84–6.75 (m, 2H), 5.64 (s, 2H), 4.54 (s, 2H), 3.78 (s, 2H), 3.10–2.67 (m, 2H), 2.62–2.23 (m, 8H); ^{13}C-NMR (75 MHz, $CDCl_3$) δ: 162.7, 158.9, 151.0, 144.5, 144.2, 136.3, 132.4, 132.3, 131.3, 130.0, 129.4, 123.1, 118.1, 113.2, 111.6, 111.4, 104.3, 104.1, 103.8, 72.1, 62.2, 56.3, 53.8, 52.7, 52.6, 40.9; IR (KBr): 3405, 2925, 2817, 2231, 1614, 1497, 1421, 1322, 1271, 1206, 1136, 1047, 1008, 963, 850, 785, 736, 681, 653 cm^{-1}; ESI-MS, *m/z* calcd. for $C_{26}H_{27}F_2N_9O$ 519.2, found $[M+H]^+$ 520.4.

1-(1H-1,2,4-Triazol-1-yl)-2-(2,4-difluorophenyl)-3-(4-((1-(3-cyanobenzyl)-1H-1,2,3-triazol-4-yl)methyl) piperazin-1-yl)-2-propanol (**1q**). Oil, yield, 63%; ^{1}H-NMR (300 MHz, $CDCl_3$) δ: 8.07 (s, 1H), 7.80 (s, 1H), 7.66–7.62 (m, 2H), 7.53–7.48 (m, 4H), 6.84–6.75 (m, 2H), 5.94 (s, 2H), 4.57 (s, 2H), 4.03 (s,2H), 3.23–3.09 (m, 2H), 2.63–2.24 (m, 8H); ^{13}C-NMR (75 MHz, $CDCl_3$) δ: 162.8, 158.9, 151.0, 144.6, 144.4, 136.3, 132.3, 132.2, 131.3, 130.0, 129.3, 123.1, 118.0, 113.3, 111.8, 111.7, 104.6, 104.2, 103.9, 72.2, 62.2, 56.3, 53.8, 52.8, 52.3, 40.9; IR(KBr): 3406, 2926, 2819, 2230, 1616, 1499, 1422, 1323, 1272, 1207, 1137, 1049, 1009, 964, 851, 786, 738, 680, 654 cm^{-1}; ESI-MS, *m/z* calcd. for $C_{26}H_{27}F_2N_9O$ 519.2, found $[M+H]^+$ 520.5.

1-(1H-1,2,4-Triazol-1-yl)-2-(2,4-difluorophenyl)-3-(4-((1-(4-cyanobenzyl)-1H-1,2,3-triazol-4-yl)methyl) piperazin-1-yl)-2-propanol (**1r**). Oil, yield, 67%; ^{1}H-NMR (300 MHz, $CDCl_3$) δ: 8.10 (s, 1H), 7.77 (s, 1H), 7.69–7.64 (m, 3H), 7.50–7.32 (m, 3H), 6.83–6.74 (m, 2H), 5.68 (s, 2H), 4.53 (s, 2H), 3.72 (s, 2H), 3. 08–2.64 (m, 2H), 2.61–2.23 (m, 8H); ^{13}C-NMR (75 MHz, $CDCl_3$) δ: 162.6, 158.8, 150.9, 144.6, 144.3, 136.2, 132.4, 132.2, 131.2, 130.0, 129.4, 123.0, 117.9, 113.2, 111.7, 111.3, 104.7, 104.3, 103.8, 72.1, 62.1, 56.2, 53.8, 52.8, 52.7, 40.8; IR(KBr): 3407, 2927, 2817, 2231, 1617, 1497, 1423, 1325, 1272, 1208, 1135, 1048, 1008, 963, 852, 787, 737, 681, 655 cm^{-1}; ESI-MS, *m/z* calcd. for $C_{26}H_{27}F_2N_9O$ 519.2, found $[M+H]^+$ 520.3.
